# The Contribution of Free Sugars to Energy Intake in Mid to Late Childhood: Comparisons Between Nutrient and Food Group Intakes and Antecedents of Diets High and Low in Free Sugars

**DOI:** 10.3390/nu16234192

**Published:** 2024-12-04

**Authors:** Pauline M. Emmett, Caroline M. Taylor

**Affiliations:** Centre for Academic Child Health, Bristol Medical School, University of Bristol, Canynge Hall, 39 Whatley Rd, Bristol BS8 2PS, UK; caroline.m.taylor@bristol.ac.uk

**Keywords:** healthy dietary pattern, core foods, discretionary foods, sugar-sweetened soft drinks, fruit juice, vegetables, protein, dietary fibre, ALSPAC

## Abstract

**Background/Aims:** High intakes of free sugars may have negative effects on health perhaps associated with their effect on nutrient and food group intakes. The primary aim of the study was to compare nutrient and food group intakes between children with habitual high or low intakes of free sugars to identify which foods could be targeted to improve the diets of children consuming excess free sugars. The secondary aim was to assess antecedents for a child being in the high free sugars group compared with the low to identify the age at which an intervention would be most effective. **Methods:** The Avon Longitudinal Study of Parents and Children collected 3-day food records from children at ages 7, 10, and 13 years. Increments of 5% of energy from free sugars (%E-FS) were calculated. Two groups of children were identified: those consuming ≤15%E-FS each time (Low-FS) and those consuming >20%E-FS each time (High-FS). Their mean daily nutrient and food group intakes were compared at each age using ANOVA. Antecedents of being in these two FS groups were tested using regression models. **Results:** At each age, 70% of children consumed >15%E-FS with one-third >20%E-FS. Data were available for 4723 children at all three ages, and the diets of the 456 children with Low-FS intakes were compared with 330 children with High-FS intakes at each age. Energy intakes were higher in High-FS than Low-FS at each age, but protein, fat, starch, and fibre intakes were lower. Several micronutrient intakes (e.g., calcium, zinc, selenium, and retinol) were also lower. The High-FS group ate more confectionery and other sweet foods than the Low-FS group as well as six times more sugar-sweetened soft drinks (SSSD) and four times more fruit juice. However, the High-FS group consumed less bread, fat spreads, milk, and vegetables than the Low-FS group. Being in the High-FS group compared with Low-FS group was not associated with maternal education, age, or breastfeeding duration. It was more likely if the child was difficult to feed at 15 months or a picky eater in preschool years and if a dietary pattern low in nutrient-dense core foods and high in nutrient-poor discretionary foods was being consumed at 2 and/or 3 years of age. **Conclusions:** Children who habitually ate a High-FS diet in mid-late childhood consumed larger amounts of SSSD and fruit juice and less of some core foods than Low-FS consumers. This type of dietary pattern was already evident at 2 years of age and was associated with being a picky eater. To reduce children’s intake of free sugars, support for parents to introduce a healthy balanced diet should be provided in the first 2 years of a child’s life.

## 1. Introduction

Both WHO and the UK Scientific Advisory Committee on Nutrition (SACN) recommend that ‘free sugars’ should be the category of dietary sugars used when assessing health risks [1,2]. Their definition of free sugars includes all mono- and disaccharides in the diet except those that are present in milk and intact whole fruit and vegetables. This definition means that sugars in 100% fruit and vegetable juices and purees are part of free sugars [3]. In the UK, ‘non-milk extrinsic sugars’ has been used as a category for measuring sugars: it includes the sugars in 100% fruit and vegetable juices and encompasses very similar intakes to free sugars [1]. In 2022, the European Food Safety Authority (EFSA) published a scientific opinion regarding the safety of dietary sugars [4], finding evidence of moderate certainty for a positive and causal relationship between intakes of free sugars and obesity and dyslipidaemia. However, they could not find sufficient evidence to provide a tolerable level of intake and concluded that the intake of free sugars should be as low as possible in the context of a nutritionally adequate diet. A very long-term study using quasi-experimental data from the UK at the end of wartime sugar and sweets rationing in September 1953 has contributed to the evidence of the detrimental effect of sugar intake. Children who were rationed in the first 2 years of life consumed less than half the amount of sugar than those who were not, and the lower sugar intake was associated with comparatively low incidence of diabetes and hypertension in adult life [5].

There are concerns that high intakes of free sugars may lead to the displacement of nutrient-dense foods from the diet, thus reducing its overall nutrient content and possibly leading to inadequate intakes of some nutrients. However, a review of the literature found inconsistent results that were very dependent on the dietary data collection methodology and the statistical approach used [6]. The misreporting of energy intake was a particular problem.

The median intake of free sugars (SACN definition) [1] in the UK National Diet and Nutrition Survey (NDNS) 2014–2016 was 13% of energy in children aged 4–10 and 11–18 years [7]. Only 3% of 4–10 and 6% of 11–18-year-olds in NDNS had intakes of ≤5% of energy from free sugars as recommended by SACN [1], while 28% and 26%, respectively, were below 10% (WHO recommendation) [2]. In the younger NDNS age group, 14.6% of free sugars came from confectionery (including chocolate), 10.1% from sugar-sweetened soft drinks (SSSD), and 10.9% from fruit/vegetable juices. In the older age group, these were 11.9%, 22.7%, and 10.1%, respectively. However, NDNS is a cross-sectional survey; therefore, although it shows a large difference in SSSD consumption between younger and older children, it cannot differentiate between an age effect and a cohort effect. Further insight would be gained by following a cohort of children as they age.

Very few studies have followed children longitudinally and obtained dietary information from them at intervals during their childhood. One such study is the Avon Longitudinal Study of Parents and Children (ALSPAC), which followed a cohort of children from birth to adolescence and collected food records using a comparable method to NDNS. It is likely that some children, having started on a diet high in free sugars at a young age, continue the same type of diet throughout childhood and that other children follow a low-free sugars diet during that same period. This study used dietary data collected by the ALSPAC when the study children were aged 7, 10, and 13 years to identify two groups of children: those with high intake of free sugars at each age and those with low/moderate intake of free sugars at each age. The primary aim of the study is to compare the nutrient and food group intakes between these two groups of children to identify which foods could be targeted to improve the balance of the diet in high-free sugars consumers. The secondary aim is to explore the antecedents of a child consuming a diet high in free sugars compared with a diet low in free sugars to identify the age at which an intervention should be targeted.

## 2. Methods

Participants were the offspring of women taking part in ALSPAC, a prospective birth cohort study that recruited pregnant women resident in a defined part of southwest England around the city of Bristol. The women had an expected delivery date between April 1991 and December 1992 (14,541 pregnancies were enrolled) [8]. Of the initial pregnancies, there were 14,062 live births (13,988 children who were alive at 1 year of age) [9], and these were followed throughout childhood. When the children were aged 7 years, they were invited to attend a research clinic, and eligible children who had not joined the study at the beginning were invited: 456 new eligible children were recruited at age 7 years. The ALSPAC study website contains details of all data that are available through a fully searchable data dictionary and variable search tool (http://www.bristol.ac.uk/alspac/researchers/our-data/, accessed 2 December 2024).

### 2.1. Dietary Assessment

Diet was assessed using a parental completion food record at 7 years. The children themselves were sent food records at 10 and 13 years of age and asked to complete them with parental help. The structured record covered all foods and drinks consumed by the child over three individual days (preferably 1 weekend day and 2 weekdays which did not need to be consecutive). There were prompts in the records to encourage correct recording and household measures were used to describe portion sizes. At the 10- and 13-year clinics the child and parent were interviewed by a nutrition fieldworker to obtain further details about the foods consumed. At 10 and 13 years, if no diary had been brought to the clinic, the nutrition fieldworker carried out a 24-h recall for the previous day’s diet with the child and parent. A short questionnaire accompanied the food record and gained further details to aid interpretation of the food record. For example, the volume of the usual cup used for drinks was recorded [10].

The food records were transformed into weights and codes linkable to the nutrient content corresponding to each of the foods and drinks consumed obtained from UK food composition tables [10]. Food weights were allocated based on described portion sizes; if the description was inadequate portion size was based on weighed intake data from a national sample of similar aged children. Mean daily energy and nutrient intakes and weights of foods and drinks consumed were calculated by averaging the 3 days of food records. Foods/drinks were aggregated into food groups based on core nutrient-rich foods, recommended as part of a balanced diet and discretionary nutrient-poor foods, usually defined by their high fat and/or sugar content (Supplementary Table S1). Intakes from dietary supplements were not included in the nutrient calculations.

A measure of free sugars intake was calculated by deducting the sugars from milk, fruits and vegetables from total sugars. The sugar contents of fruit juices, honey, and syrup were included in free sugars [1]. Dietary fibre was measured as non-starch polysaccharides (NSP): this does not include resistant starch or lignin substances so is about 25% lower than AOAC-measured fibre [1].

For energy-containing nutrients, the percentage energy contribution was calculated. Individuals were grouped according to their percentage energy intake from free sugars (%E-FS) in 5% increments. Initially, seven groups were defined (≤5%, >5 to 10%, >10 to 15%, >15 to 20%, >20 to 25%, >25 to 30%, >30%) at each age of dietary assessment. From children who had provided food records at each age, two groups were identified: those consuming 15% or less %E-FS at each of the three ages (low free sugars, Low-FS) and those consuming more than 20%E-FS at each age (high free sugars, High-FS). The dietary nutrient and food group intakes of children in these two groups were compared.

### 2.2. Misreporting of Dietary Intake

Misreporting of energy intake (EI) was assessed by an individualised method using sex, age, and body weight at assessment with an increment for growth and a standard level of physical activity [11]. The ratio of reported EI to estimated energy requirement (EER) was calculated (EI:EER) at each age. Individual EERs were estimated using equations from the FAO/WHO/UNU Expert Consultation report on human energy requirements [12]. A 95% confidence interval (CI) for the accuracy of EI:EER accounted for the variations inherent in the methods. At each age children who had reported plausible energy intakes were identified.

### 2.3. Dietary Reference Values

Dietary reference values for food energy and nutrients for the UK population were used to assess the adequacy of the diet at each age [13] by computing a Mean Adequacy Ratio (MAR) for 12 selected nutrients (protein, potassium, calcium, iron, zinc, selenium, iodine, riboflavin, folate, vitamin D, polyunsaturated fatty acids, and fibre) [14]. The SACN report *Carbohydrates and Health* in 2015 [1] recommended an NSP intake of 15 g/day for 5–10-year-olds and 19 g/day for 11–16-year-olds.

### 2.4. Background and Early Diet Variables

Data from parent-completed postal questionnaires during pregnancy and after the birth of the child were used to obtain maternal and early childhood diet variables. These included maternal age at delivery (<25, 25–30, >30 years of age) and highest educational attainment summarised as two categories: Low (No qualifications; Vocational; Ordinary Level Certificate of School Education, usually taken at 16 years of age) and High (Advanced Level Certificate, usually taken at 18 years of age; Degree). Maternal pre-pregnancy body mass index was categorised as <20, 20–24.99, ≥25 kg/m^2^, parity as 0, 1, ≥2, and birthweight as ≤2500, 2501–4000, >4000 g).

Early feeding information was collected on the age of introduction of complementary foods (categorised as 0–3, 3–5, >5 months), duration of breastfeeding (none, <3 months, 3–5 months, ≥6 months), age of introduction of lumpy/chewy foods (<6, 6–9, ≥10 months), and feeding difficulties at aged 15 months (Yes, No). Dietary patterns were extracted using principal components analysis from a questionnaire when the children were 2 years of age [15]. Three dietary patterns described the data: ‘Family foods’ (high loadings on bread, breakfast cereals, biscuits, meat, fish, cheese, puddings, potatoes, and cooked vegetables), ‘Sweet & Easy’ (sweets, chocolate, carbonated SSSD, and flavoured milk drinks, as well as crisps, potatoes, baked beans, peas, soup, tea/coffee), and ‘Healthy’ (fresh fruit, raw and cooked vegetables, fish, eggs, cheese, legumes, nuts, and fruit juice). Dietary patterns were extracted again from a questionnaire when the children were 3 years of age; however, this time, four patterns described the data [16]. These were: ‘Junk’ (high positive loadings on white bread, sausages/burgers, chips, pizza, fried foods, crisps, sweets, biscuits, chocolate, carbonated SSSD), ‘Healthy’ (fish, eggs, cheese, rice, pasta, legumes, fresh fruit, cooked and raw vegetables, vegetarian-style foods, fruit juice), ‘Traditional’ (positive loadings on meat, poultry, potatoes (not chips), cooked vegetables and negative with vegetarian-style foods), ‘Snacks’ (cheese, bread, breakfast cereals, puddings, cakes/buns, biscuits, chocolate, crisps, fresh fruit, and SSSD). All dietary pattern scores were analysed as quartiles with the 4th quartile representing the highest scores on each pattern.

The number of siblings present in the family was assessed using a questionnaire when the child was 6 years old (none, older + younger, younger only, older only). Preschool picky eating was identified from questionnaires administered when the children were 2, 3, 4.5, and 5.5 years old. Picky eating was classified from responses to the following question: ‘Does your child have definite likes and dislikes as far as food is concerned?’ with three categories of answer: No/Yes, quite choosy/Yes, very choosy. Very picky eaters were children who were very choosy at two or more time points, Somewhat picky eaters were children who were choosy at some but not all time points [17].

### 2.5. Statistical Analyses

Statistical analysis was carried out with SPSS v23 (IBM Corp., Chicago, IL, USA).

The distribution of %E from free sugars at 5% intervals was determined at each age for all participants and for plausible reports only. ANOVA was used to compare nutrient and food group intakes, and the MAR for each age between the diets of the Low-FS and High-FS groups only.

#### Assessment of the Antecedents of Sugar Intake

A minimally adjusted logistic regression model predicting being in the High-FS group compared with the Low-FS group included demographic and perinatal variables (age and education status of the mother, pre-pregnancy BMI group, parity, sex of the child, and birth weight (categorical)) as predictors and was the basis for the life stage models. Model 1 introduced additional covariates occurring in the first 2 years of life: age at introduction of complementary foods, duration of breastfeeding, age at introduction of lumpy foods, difficulty feeding at 15 months and dietary patterns at age 2 years. Model 2 used the minimal model with additional covariates occurring at 3–6 years of age: whether the child had been a picky eater, whether they had older, younger, or no siblings at age 6 years, and their dietary patterns at age 3 years.

## 3. Results

**Figure 1:**
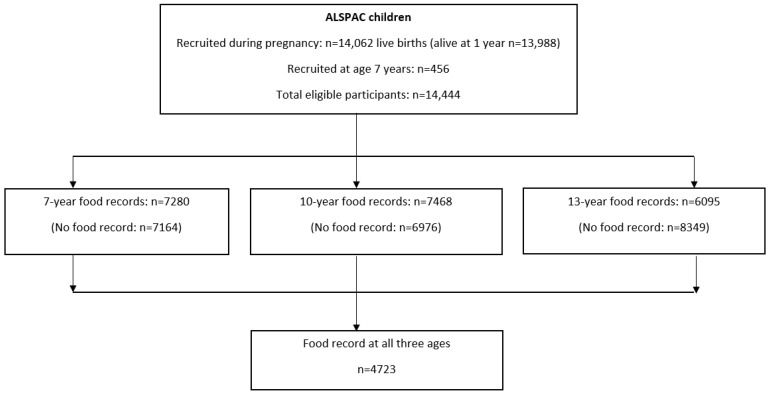
Study flow diagram for children taking part in the Avon Longitudinal Study of Parents and Children (ALSPAC). The present study uses data from children with food records at 7, 10, and 13 years of age and those with a food record at all three ages. ‘No food record’ includes child death, withdrawals, and food records not returned.

A flowchart (Figure 1) shows the recruitment process for the participating children with dietary records at ages 7, 10, and 13 years and those who provided records at all three ages. At each age, children were excluded if they did not provide a food record. At the next stage, children who did not provide a food record at all three ages were excluded. Supplementary Table S2 compares the background demographic variables of the children with food records at all ages and those without complete dietary data. Compared with the included children, those who were excluded were slightly more likely to be male and had a higher mean birthweight; their mothers were more likely to be in the low education group, to have been under 25 years of age at their child’s birth and had a slightly higher mean pre-pregnancy BMI.

The numbers of children with dietary data at 7, 10, and 13 years of age and the distribution of %E-FS are shown in Table 1. Misreporting of dietary intake was common and increased with age: the distribution of %E-FS was repeated in plausible reporters only (Supplementary Table S3). At 7 years of age, the spread of intakes in the plausible reporters was the same as in the full cohort; however, at 10 and 13 years, the spread had moved towards higher intakes of %E-FS in the plausible reporters compared with the whole cohort.

Very few children were consuming ≤5% or >30%E-FS. The most common amount of energy supplied by free sugars at all three ages was between 15% and 20% (Table 1), covering about one-third of children, with the overall mean intake being about 17%E-FS. At each age, the children were divided into approximate thirds by %E-FS intake: ≤15% (Low-FS), >15–20%, >20% (High-FS). Of the children who provided food records at each of the three ages (n = 4723), 456 (9.6%) consumed ≤15%E-FS each time, while 330 (7.0%) consumed >20%E-FS each time.

The diets of the children in High-FS and Low-FS groups were compared for nutrient and food group intakes. Table 2 shows the differences in nutrient content of the diets of the two groups. At each age, energy intake was more than 5% higher in the High-FS than in the Low-FS group. Although the contribution to energy from overall carbohydrates was higher in High-FS, this masked a lower contribution from starch and intrinsic sugars. The contributions to energy from protein and all three types of fat were also lower in High-FS than in Low-FS at each age. The High-FS group consumed 13% less dietary fibre at each age than the Low-FS group. Nutrients such as calcium, zinc, selenium, retinol, vitamins D and E, and riboflavin were consumed in lower amounts in High-FS than Low-FS diets, and the overall MAR of the diet was also lower at each age. The nutrients that were most likely to be inadequate in both groups at each age were vitamin D and dietary fibre.

At each age, the High-FS group ate more than double the amount of confectionery than the Low-FS group and higher amounts of other sweets foods (Table 3). They consumed six times more SSSD and four to five times more fruit juice than the Low-FS group, although a very similar amount of diet soft drinks. At each age, when added together, the total amount of soft drinks was more than twice as high in High-FS than in Low-FS (at 13 years: 884 g and 338 g, respectively). However, the High-FS group ate smaller amounts than the Low-FS group of some core foods at each age: 20–30% less bread and fat spread (butter or margarine), 33% less milk, and 33% less vegetables. At some ages, they also ate less potatoes (plain and with fat), less fruit, less fish, or less meat products. These differences in food items eaten directly account for some of the differences in nutrient intakes: higher vitamin C intake from SSSD and fruit juice and lower calcium intake from lower milk consumption in the High-FS group.

### Antecedents of Intakes of Free Sugars

In the regression analysis, background variables were investigated first: few associations were found with being in the High-FS compared with the Low-FS group, and none with maternal education, maternal age, or pre-pregnancy BMI group (Supplementary Table S4). Being of low birthweight lowered the odds of being in the High-FS group (*p* = 0.036) and being female weakly decreased the odds (*p* = 0.066)

For feeding in the first 2 years of life, there was no association with age at the introduction of complementary foods or breastfeeding duration (Table 4). However, if the child had been introduced to lumpy/chewy foods before 6 months of age they were less likely to be in the High-FS group later. Difficulty in feeding the child at 15 months of age was associated with a 77% increase in the odds of the child being in the High-FS group later. The type of diet eaten by the child at age 2 years was also associated with their free sugars intake in mid/late childhood. There was an association with the Healthy dietary pattern score such that being in the third or fourth quartile was associated with less than half the odds of being in the High-FS group (*p* = 0.008 and 0.002, respectively), while being in the third or fourth quartile of the Sweet & Easy dietary pattern was associated with approximately double the odds of being the High-FS group (*p* = 0.015 and 0.001, respectively). Being in the second quartile of the Family Foods dietary pattern at age 2 years was associated with being in the High-FS group (about twice as likely, *p* < 0.016); however, there was no evidence of associations for the third or fourth quartile.

Some feeding experiences between 3 and 6 years of age were also associated with later free sugars intake (Table 5). Children who were picky eaters between 2 and 5 years had greater odds of being in the High-FS group (nearly double for very picky children (*p* = 0.006) compared with never picky children). There was some evidence that having both older and younger siblings was protective of being a High-FS consumer compared with being an only child. Being in the third or fourth quartile for the score on the Healthy dietary pattern at 3 years more than halved the odds of being in the High-FS group (*p* = 0.003 and <0.001, respectively) compared with being in the first quartile. For the Snack dietary pattern found at 3 years the score was associated with High-FS in the opposite direction, the highest quartile in the Snack dietary pattern score was associated with three times higher odds of being in the High-FS group (*p* < 0.001). Scores on the Junk or the Traditional dietary patterns found at this age were unrelated to the FS groups.

## 4. Discussion

In mid-late childhood, at ages 7, 10, and 13 years, 70% of children in ALSPAC were consuming >15% of their energy intake from free sugars. A group of the children who had habitual high free sugars intakes were identified and compared with a group of the children with habitual low to moderate intakes. At each of the three ages the High-FS consumers had higher energy intake but lower intakes of some key nutrients than the Low-FS consumers including lower protein, dietary fibre, calcium, zinc, and vitamin D. There were large differences in the amounts of SSSD, fruit juice, and confectionary consumed but similar amounts of diet soft drinks between the two groups. The High-FS consumers ate a greater amount of discretionary foods and a lesser amount of core foods including less bread, milk, and vegetables than the Low-FS consumers. We have found a deficit in some key nutrients and an excess of free sugars in the diets of the High-FS group. This type of diet has been associated with diabetes, hypertension, dyslipidaemia, and obesity in adults [4,5] following these children into adulthood could be informative. The antecedents of High-FS consumption became evident early in life with feeding difficulties at 15 months of age and preschool picky eating problems associated with increased odds. By the age of 2 years and again at 3 years, there was evidence that the children who were in the High-FS group by age 7 years were more likely to have consumed a less healthy diet than children who were low to moderate FS consumers. This indicates that interventions to reduce the intakes of free sugars in childhood need to start before 2 years of age and to encourage the consumption of core foods such as bread, milk, and vegetables in place of discretionary foods.

Intakes of free sugars overall in this study were very similar to those of German children followed in the Donald study, using a similar dietary assessment method (median 17.3%E-FS (IQR 13.7–21.5 at age 6–10 years and 16.8%E-FS (IQR 12.7–21.4) at 11–14 years) [18]. The UK NDNS 2014–2016 found slightly lower intakes of free sugars (median 13% E-FS (IQR 10–16) at age 4–10 years and 13%E-FS (10–18) at 11–18 years) [7]. The Donald study also investigated the temporal pattern of free sugars intake in Germany, showing that mean intakes rose from 1985 to 2005 but declined quite sharply between 2010 and 2016 [18]. The ALSPAC data were collected during the period of rising intakes (1998–2006), while the NDNS data were collected (2014/2016) when intakes in Germany were decreasing.

Our finding of a worse nutrient profile in the High-FS group compared with the Low-FS was similar to that found in a Norwegian study of ‘added sugars’ intake in groups of children aged 4, 9, and 13 years who completed 4-day food diaries in 2000 [19]. The mean %E from added sugars increased by age (15.1, 16.8, and 18.4%E, respectively). At each age, comparisons were made across quartiles of intake of added sugars. There were no differences in energy intake between any of the quartiles in any of the age groups. However, similar to our study, %E from protein and fat was lower and carbohydrate higher as added sugars quartile increased at each age. Fibre, calcium, retinol, vitamin D, and riboflavin intakes were assessed and showed a decrease as added sugars intake quartile increased at each age. Some food group intakes were investigated with vegetables, fruit, potatoes, and dairy products showing a negative gradient and SSSD with a positive gradient similar to our study. An Australian study of 6150 adults, assessing diet using two 24-h recalls, found increased odds of a poor micronutrient profile with intakes of free sugars above 25%E [20]. This was associated with increased consumption of discretionary foods, such as SSSD and confectionary, and decreased consumption of core foods such as vegetables, fruit, fish, and meat, a similar finding to our study.

As confirmed in the present study, SSSD, fruit juice, and confectionery are major sources of free sugars in mid-childhood in many countries. For example, the European Childhood Obesity Project (CHOP) followed children in five different European countries from ages 1–8 years collecting 3-day food records at eight ages, they assessed total sugar intake but within that published confectionary and SSSD results separately [21]. Intakes of confectionary rose from 5% to 24% and SSSD from 2% to 6.7% of total sugars intake as the children aged. Belgian children had the highest %E from confectionary, with Italian children having the highest from SSSD. A study in Canada of children 1–8 and 9–18 years old using 24-h recalls looked at the % of total sugars from various sources of sugars [22]. For 1–8-year-olds, fruit juice provided 14.6%, confectionary 8.7%, and SSSD 3.6% of total sugars; in the older age group (9–18 years), SSSD provided 14.3%, confectionary 10.3%, and fruit juice 9.1%, showing the rise in the importance of SSSD as children grow into adolescence.

In our study, we found that SSSD contributed six times greater amounts to the intake of free sugars in the High-FS than in the Low-FS group. Evidence is accumulating that high consumption of SSSD, and fruit juice may be detrimental to health [23,24]. Since the time of our data collection, the UK Government has implemented a Soft Drinks Industry Levy (SDIL) in April 2018 [25], which has had the effect of decreasing the amount of free sugars added to SSSD [26,27]. Had this initiative been in place when our data were collected, it would likely have led to a lower intake of free sugars in these children [28].

Turning to the antecedents of habitual High-FS consumption, we found that consumption of a poor dietary pattern, high in sweets, chocolate, and SSSD, was already evident by the age of 2 years in children who were in the High-FS group later. This is of concern because high sugar intake in early life has been associated with an increased risk of diabetes and hypertension in later life [5]. The High-FS group were also more likely to have been difficult to feed at 15 months and to have been preschool picky eaters. In previous work, using ALSPAC data, we explored this behaviour in depth and concluded that parents need support in feeding their child in the first years of life to avoid these outcomes [29,30]

Habituation to consuming a high sugar diet at such an early age is likely to lead to a lifetime of this type of diet [31]. A systematic review looked at the socio-cognitive determinants of SSSD consumption in young people (mean age 15 years) [32]. It found that the strongest determinants of the amount consumed were habit, intention to consume, and a positive attitude to SSSD; for frequency of consumption the strongest determinants were intention to consume and friends’ attitudes to SSSD. This suggests that early consumption of these drinks is likely to lead to continued consumption. In our study, by the age of 2 years and again at 3 years, the consumption of SSSD was more likely in the dietary patterns associated with being in the High-FS group later in childhood.

A separate analysis of ALSPAC dietary data found that early exposure to SSSD, particularly cola drinks, before 2 years was associated with greater adiposity at 24 years of age compared with non-consumers, and that cola drinking was a marker for consumption of burgers/sausages, pizza, French fries, fried food, meat, chocolate, sweets, and avoidance of fresh fruit [33]. A study in the USA assessed the dietary patterns of children at 9 months and again at 6 years of age and found that greater intakes of SSSD, sweet dessert foods, and French fries at 9 months were associated with greater intakes of these foods and with greater likelihood of being overweight at 6 years [34]. These studies concluded that attention should be paid to diet in very young children, and this should include all foods, not just SSSD. In our study, we found that the adoption of a diet characterised by sweets, chocolate, carbonated SSSD, and flavoured milks, as well as crisps, potatoes, baked beans, peas, and soup by the age of 2 years was associated with a greater likelihood of being in the High-FS group.

The strengths of this study are in the availability of longitudinal data from birth to 13 years of age, with dietary data collected at intervals by questionnaire in early childhood and from detailed food records in mid and late childhood. Information on other feeding behaviours was also collected contemporaneously. Children who were habitually High-FS or Low-FS consumers were selected to be studied more closely to identify clear differences between the groups that might be helpful in designing interventions to reduce intakes of free sugars. However, this study’s findings are limited to Western-style diets with bread and milk as core foods, these are characteristic of diets in much of Europe, Australia, and the USA. For countries where the core foods are rice and other cereals and/or do not consume cows’ milk, it is likely that other foods are displaced when free sugars are consumed in excess. Further longitudinal research in areas where diets are based on different core foods and free sugars are consumed frequently would be informative.

The decision to select habitual High-FS or Low-FS consumers only is also one of the limitations of this study, as this meant that food records needed to be available at all three ages and be either high or low at each age, thus limiting the numbers of children available for the in-depth part of the study. Although these children are less representative of the whole population than the original ALSPAC cohort, we achieved our aim of obtaining clear differences between the two types of diet that will inform interventions. Attrition in longitudinal studies is inevitable and increases with time, and dietary data collection has well-recognised biases such as the misreporting of energy intake. We assessed the plausibility of diet reporting by a standard method and found that, at 10 and 13 years of age, higher intakes of free sugars were more likely in plausible reporters than misreporters. Thus, using plausible and misreporters together in the main analysis was likely to underestimate the effect sizes found.

## 5. Conclusions

We found that 70% of children in mid to late childhood were consuming more than 15% of their energy intake from free sugars. We studied two groups of these children in-depth, namely, habitual High-FS and habitual Low-FS consumers. The High-FS group had, on average, lower intakes of some key nutrients than the Low-FS group. They consumed much greater amounts of SSSD, fruit juice, and confectionery but less of some core foods such as bread, milk, and vegetables. When investigating the antecedents of the diets in these two groups, we found that there were already dietary differences at 2 years of age and that High-FS consumers were more likely to be difficult to feed and choosy with food from an early age than Low-FS consumers. To reduce free sugars intake, support should be given to parents in the first 2 years of a child’s life around introducing their child to a balanced diet with a full range of core foods. Advice should promote bread, milk, and vegetables as core foods and replace SSSD and fruit juice with water as a drink.

## Figures and Tables

**Table 1:** Distribution of percent energy contribution of free sugars from 3-day food records collected at ages 7, 10, and 13 years from children taking part in the Avon Longitudinal Study of Parents and Children between 1999 and 2008.

Age at Food Record (Years)	≤5%	>5–10%	>10–15%	>15–20%	>20–25%	>25–30%	>30%	Total
7	15 (0.2%)	458 (6.3%)	2020 (27.7%)	2708 (37.2%)	1493 (20.5%)	464 (6.4%)	122 (1.7%)	7280
10	48 (0.6%)	545 (7.3%)	1978 (26.5%)	2523 (33.8%)	1659 (22.2%)	558 (7.5%)	157 (2.1%)	7468
13	119 (2.0%)	758 (12.4%)	1776 (29.1%)	1823 (29.9%)	1011 (16.6%)	422 (6.9%)	186 (3.1%)	6095

**Table 2:** Comparison of energy and nutrient intakes and diet adequacy at ages 7, 10, and 13 years between children who habitually consumed either a diet low in energy from free sugars (n = 456) or one high in energy from free sugars (n = 330).

Nutrients per Day	7 Years		10 Years		13 Years	
	Low-FS	High-FS	Low-FS	High-FS	Low-FS	High-FS
Free sugars						
E%	11.6 (11.3, 11.8)	24.8 (24.4, 25.2) ***	11.2 (11.0, 11.5)	24.8 (24.4, 25.3) ***	10.3 (10.0, 10.6)	25.7 (25.1, 26.3) ***
g	51 (49, 52)	115 (112, 117) ***	54 (52, 55)	125 (122, 128) ***	52 (50, 54)	139 (134, 145) ***
Energy	7.01 (6.88, 7.13)	7.40 (7.27, 7.54) ***	7.63 (7.49, 7.77)	8.06 (7.90, 8.22) ***	7.93 (7.73, 8.13)	8.69 (8.44, 8.94) ***
Protein						
E%	14.5 (14.3, 14.6) ***	11.8 (11.6, 12.0)	15.0 (14.7, 15.2) ***	12.1(11.9, 12.4)	15.7 (15.4, 16.0) ***	12.8 (12.5, 13.1)
g	59 (58, 61) ***	51 (50, 52)	67 (65, 68) ***	57(56, 59)	72 (71, 74) ***	65 (63, 67)
Total fat						
E%	37 (37, 38) ***	33 (33, 34)	38 (37, 38) ***	33 (33, 34)	37 (36, 37) ***	31 (31, 32)
g	71 (69, 72) **	66 (65, 68)	78 (76, 80) ***	73(71, 75)	80 (77, 82) *	75 (72, 77)
Saturated fat (E%)	15.0 (14.7, 15.3) ***	13.6 (13.3, 13.9)	14.4 (14.1, 14.7) ***	13.3 (13.0, 13.6)	13.9 (13.6, 14.2) ***	12.0 (11.7, 12.4)
Monounsaturated fat (E%)	12.4 (12.3, 12.6) ***	11.0 (10.8, 11.1)	12.8 (12.6, 13.0) ***	11.3 (11.1, 11.5)	12.5 (12.2, 12.7) ***	10.5 (10.3, 10.7)
Polyunsaturated fat (E%)	5.9 (5.7, 6.0) ***	4.9 (4.7, 5.0)	6.5 (6.3, 6.7) ***	5.1 (5.0, 5.3)	6.5 (6.3, 6.7) ***	5.0 (4.8, 5.2)
Carbohydrate						
E%	48 (48, 49)	55 (55, 56) ***	47 (47, 48)	55 (54, 55) ***	48 (47, 48)	56 (55, 56) ***
g	212 (208, 216)	255 (250, 260) ***	226 (221, 230)	275 (269, 280) ***	235 (229, 241)	302 (293, 310) ***
Starch (E%)	28 (28, 29) ***	24 (23, 24)	29 (29, 30) ***	24 (24, 25)	30 (30, 31) ***	25 (24, 25)
Intrinsic sugars (E%)	8.2 (7.9, 8.6) ***	6.2 (5.9, 6.5)	6.4 (6.1, 6.7) ***	4.9 (4.6, 5.2)	6.4 (6.0, 6.7) ***	4.6 (4.3, 5.0)
Dietary fibre (NSP) (g)	11.4 (11.0, 11.7) ***	9.9 (9.6, 10.2)	12.5 (12.1, 12.9) ***	10.9 (10.5, 11.2)	13.7 (13.2, 14.2) ***	12.0 (11.5, 12.5)
Sodium (mg)	2406 (2351, 2461) ***	2180 (2125, 2235)	2742 (2675, 2809) ***	2416 (2350, 2481)	2781 (2697, 2865) ***	2511 (2424, 2598)
Potassium (mg)	2285(2229, 2341) **	2169 (2109, 2229)	2461 (2404, 2519)	2401 (2338, 2464)	2618 (2543, 2694)	2691 (2605, 2777)
Calcium (mg)	864 (836, 891) ***	730 (704, 755)	864 (833, 897) ***	757 (729, 785)	910 (873, 947) ***	800 (766, 833)
Iron (mg)	8.4 (8.2, 8.6)	8.4 (8.1, 8.6)	9.1 (8.9, 9.4)	8.9 (8.7, 9.1)	10.0 (9.7, 10.3)	10.1 (9.7, 10.4)
Zinc (mg)	6.6 (6.4, 6.7) ***	5.6 (5.5, 5.8)	7.2 (7.0, 7.4) ***	6.2 (6.0, 6.4)	8.0 (7.7, 8.2) ***	7.1 (6.9, 7.4)
Iodine (μg)	163 (155, 170) ***	137 (130, 144)	134 (127, 140)	125 (120, 131)	148 (140, 156)	140 (132, 148)
Selenium (μg)	57 (55, 58) ***	48 (46, 50)	64(63, 67) ***	53 (52, 55)	68 (66, 70) ***	58 (55, 60)
Retinol (μg)	434 (390, 478) *	359 (307, 410)	446 (395, 498) **	333 (281, 385)	376 (354, 398) ***	296 (273, 318)
Carotene (μg)	1905 (1769, 2040)	1985 (1814, 2155)	2180 (1999, 2360)	2174 (1994, 2354)	2379 (2186, 2572)	2575 (2360, 2791)
Vitamin D (μg)	2.4 (2.3, 2.5) **	2.2 (2.1, 2.3)	2.9 (2.8, 3.0) ***	2.4 (2.2, 2.5)	2.8 (2.7, 3.0) *	2.5 (2.4, 2.7)
Vitamin E (mg)	8.4 (8.1, 8.7) **	7.6 (7.3, 8.0)	9.9 (9.5, 10.2) ***	8.3 (7.9, 8.6)	9.8 (9.4, 10.3) ***	8.6 (8.2, 9.0)
Riboflavin (mg)	1.8 (1.7, 1.8) ***	1.6 (1.5, 1.6)	1.7 (1.6, 1.7) *	1.6 (1.5, 1.6)	1.9 (1.8, 1.9) **	1.7 (1.6, 1.8)
Folate (μg)	201 (195, 207)	200 (192, 208)	214 (207, 220)	209 (201, 216)	236 (228, 244)	234 (225, 244)
Vitamin C (mg)	57 (54, 61)	118 (110, 127) ***	64 (60, 68)	125 (117, 133) ***	74 (69, 79)	152 (141, 162) ***
Mean Adequacy Ratio (MAR)	0.87(0.86, 0.87) ***	0.84 (0.83, 0.85)	0.89 (0.88, 0.89) ***	0.86 (0.85, 0.86)	0.82 (0.81, 0.83) **	0.78 (0.77, 0.80)

Low free sugars group (Low-FS) consumed ≤15% of energy from free sugars at each of the three ages. High free sugars group (High-FS) consumed >20% of energy from free sugars at each of the three ages. %E, percentage contribution to total energy intake. * *p* < 0.05, ** *p* < 0.01, *** *p* < 0.001. NSP, non-starch polysaccharide (a measure of dietary fibre). MAR is based on adequate intakes of protein, polyunsaturated fatty acids, dietary fibre, potassium, calcium, iron, zinc, iodine, selenium, riboflavin, folate, and vitamin D.

**Table 3:** Comparison of food group intakes at ages 7, 10, and 13 years between children who habitually consumed either a diet high in energy from free sugars (n = 330) or one low in energy from free sugars (n = 456).

Food Groups (g/Day)	7 Years		10 Years		13 Years	
	Low	High	Low	High	Low	High
All bread	83 (79, 87) ***	65 (61, 69)	96 (91, 100) ***	71 (67, 75)	105 (100, 111) ***	75 (70, 81)
High fibre	17 (14, 19) *	12 (9, 14)	19 (16, 22) *	13 (10, 16)	29 (25, 33) ***	20 (16, 24)
Breakfast cereal	32 (30, 34)	33 (30, 35)	26 (24, 28)	26 (24, 29)	29 (26, 32)	30 (27, 34)
Sugary cereals	12 (11, 14)	17 (15, 19) ***	11 (9, 12)	14 (12, 16) *	9 (8,11)	14 (12, 17) ***
Fat spreads	13 (12, 14) ***	10 (9, 11)	15 (14, 16) ***	11 (10, 12)	15 (14, 16) ***	9 (8, 10)
Milk	322 (301, 343) ***	208 (191, 225)	266 (246, 287) ***	177 (161, 194)	264 (241, 288) ***	177 (159, 196)
Other dairy	49 (44, 53)	49 (43, 55)	47 (43, 52)	48 (42, 54)	47 (43, 52)	47 (39, 54)
Sweet foods	79 (74, 83)	110 (103, 116) ***	71 (66, 76)	107 (100, 114) ***	52 (47, 57)	100 (92, 108) ***
Biscuits	17 (16, 19)	24 (22, 26) ***	17 (15,18)	23 (21, 25) ***	14 (12, 16)	23 (20, 26) ***
Confectionery	21 (19, 22)	50 (47, 53) ***	20 (19, 22)	51 (48, 54) ***	20 (17, 22)	48 (44, 52) ***
Chocolate	11 (10, 12)	23 (21, 25) ***	12 (10, 13)	27 (24, 30) ***	11 (10, 13)	25 (22, 28) ***
Savoury snacks	17 (16, 19) *	15 (14, 16)	18 (17, 20)	17 (15, 19)	16 (14, 17)	14 (12, 16)
Meat and poultry (plain)	42 (38, 45) *	36 (32, 40)	56 (51, 61)	51 (45, 56)	76 (69, 83)	76 (68, 83)
Meat (cured, coated, pies)	38 (35, 41)	34 (31, 38)	49 (45, 53) **	40 (37, 44)	46 (41, 51)	42 (37, 47)
Fish	15(13, 17) *	12 (10, 14)	18 (16, 21) ***	11 (9, 13)	18 (15, 22)	14 (11, 17)
Vegetables	60 (55, 65) ***	43 (39, 48)	75 (70, 81) ***	52 (47, 57)	90 (84, 97) ***	60 (55, 65)
Pulses	17 (14, 19)	14 (11, 17)	25 (21, 30) *	19 (15, 23)	20 (15, 24)	17 (13, 21)
Potatoes (fried, roast)	55 (51, 59) ***	44 (40, 48)	64 (59, 69) *	57 (52, 62)	65 (59, 71)	59 (52, 65)
Plain potatoes	36 (32, 39) ***	25(22, 29)	43 (37, 47)	30 (25, 34)	46 (41, 52) *	37 (31, 43)
Pasta, rice, pizza	54 (48, 59)	48 (43, 54)	78 (72, 85)	69 (62, 77)	99 (90, 109)	93 (83, 103)
Fruit	94 (86, 102) *	81 (72, 89)	77 (69, 84)	75 (66, 84)	91 (82, 99) *	73 (63, 83)
SSSD	38 (31, 46)	268 (237, 299) ***	43 (34, 52)	290 (260, 320) ***	64 (52, 76)	378 (335, 422) ***
Diet soft drinks	289 (257, 321)	251 (217, 284)	228 (204, 252) *	184 (156, 211)	219 (189, 249)	203 (168, 239)
Fruit juice	30 (24, 35)	181 (158, 204) ***	50 (42, 57)	217 (195, 240) ***	55 (46, 63)	303 (273, 334) ***

Food group composition is described in Supplementary Table S1. Low free sugars group (Low-FS) consumed ≤15% of energy from free sugars at each of the three ages. High free sugars group (High-FS) consumed >20% of energy from free sugars at each of the three ages. SSSD, sugar-sweetened soft drinks. * *p* < 0.05, ** *p* < 0.01, *** *p* < 0.001.

**Table 4:** Model 1 (n = 693): Feeding experiences in the first 2 years of life as predictors of being in the high free sugars (High-FS) compared with the low free sugars (Low-FS) group at all three ages (7, 10, and 13 years).

Predictor Variable	Reference Category	Predictor Category	Child in High-FS Group at All Three Ages		
			OR	95% CI	*p* Value
Age solid food introduced (months)	>5	0–3 months	1.53	0.69, 3.94	0.294
		3–5 months	1.41	0.62, 3.25	0.413
Duration of breastfeeding (months)	≥6	Never	0.69	0.42, 1.14	0.149
		<3 months	0.77	0.49, 1.21	0.255
		3–5 months	0.95	0.58, 1.56	0.835
Age introduced to lumps (months)	6–11	<6 months	0.43	0.21, 0.87	0.019
		>11 months	1.38	0.72, 2.64	0.388
Difficult to feed at 15 months old	No	Yes	1.77	1.26, 2.49	<0.001
Dietary patterns at 2 years old					
Family foods	1st quartile	2st quartile	1.81	1.12, 2.95	0.016
		3nd quartile	1.25	0.76, 2.04	0.377
		4rd quartile	1.10	0.67, 1.80	0.700
Sweet & Easy	1st quartile	2st quartile	1.07	0.66, 1.73	0.796
		3nd quartile	1.82	1.12, 3.00	0.015
		4rd quartile	2.25	1.37, 3.71	0.001
Healthy	1st quartile	2st quartile	1.09	0.68, 1.75	0.717
		3nd quartile	0.51	0.32, 0.84	0.008
		4rd quartile	0.46	0.28, 0.76	0.002

Low free sugars group (Low-FS) consumed ≤15% of energy from free sugars at each of the three ages. High free sugars group (High-FS) consumed >20% of energy from free sugars at each of the three ages. Reference category: Child in low free sugars group at all three ages. Model 1 = minimal model (maternal age at delivery, highest educational attainment and pre-pregnancy body mass index, and sex and birthweight of the child) plus the variables listed above. The total variance explained is 0.132 (Nagelkerke R square).

**Table 5:** Model 2 (n = 620): Feeding experience from 3–6 years of age as antecedents of being in the high free sugars (High-FS) compared with the low free sugars (Low-FS) group at all three ages (7, 10, and 13 years).

Predictor Variable	Reference Category	Predictor Category	Child in High-FS Group at All Three Ages		
			OR	95% CI	*p* Value
Preschool picky eating	Never	Somewhat picky	2.18	1.20, 4.00	0.010
		Very picky	1.83	1.19, 2.82	0.006
Number of siblings at 6 years old	None	Both younger and older	0.48	0.23, 0.99	0.048
		Younger siblings only	0.80	0.44, 1.46	0.461
		Older siblings only	0.68	0.37, 1.24	0.206
Dietary patterns at 3 years old					
Junk	1st quartile	2nd quartile	0.94	0.57, 1.56	0.815
		3rd quartile	1.15	0.70, 1.90	0.580
		4th quartile	1.41	0.82, 2.44	0.219
Healthy	1st quartile	2nd quartile	0.88	0.54, 1.43	0.600
		3rd quartile	0.46	0.28, 0.77	0.003
		4th quartile	0.32	0.19, 0.54	<0.001
Traditional	1st quartile	2nd quartile	0.77	0.46, 1.29	0.326
		3rd quartile	0.84	0.50, 0.41	0.508
		4th quartile	0.70	0.41, 1.21	0.198
Snack	1st quartile	2nd quartile	1.22	0.73, 2.03	0.455
		3rd quartile	2.07	1.25, 3.45	0.005
		4th quartile	3.59	2.15, 5.98	<0.001

Low free sugars group (Low-FS) consumed ≤15% of energy from free sugars at each of the three ages. High free sugars group (High-FS) consumed >20% of energy from free sugars at each of the three ages. Reference category: child in low free sugars group at all three ages. Model 2 = minimal model (maternal age at delivery, highest educational attainment and pre-pregnancy body mass index, and sex and birthweight of the child) plus the variables listed above. The total variance explained is 0.209 (Nagelkerke R square).

## Data Availability

The ALSPAC study website contains details of all the data that are available through a fully searchable data dictionary and variable search tool: http://www.bris.ac.uk/alspac/researchers/data-access/data-dictionary/. ALSPAC data access is through a system of managed open access. These steps highlight how to apply for access to all ALSPAC data: (1) Read the ALSPAC access policy (http://www.bristol.ac.uk/media-library/sites/alspac/documents/researchers/data-access/ALSPAC_Access_Policy.pdf), which describes the process of accessing the data and samples in detail, and outlines the costs associated with doing so; (2) Browse the fully searchable research proposals database (https://proposals.epi.bristol.ac.uk/?q=proposalSummaries), which lists all research projects that have been approved since April 2011; (3) Submit your research proposal (https://proposals.epi.bristol.ac.uk/) for consideration by the ALSPAC Executive Committee.

## Supplementary Materials

The following supporting information can be downloaded at: https://www.mdpi.com/article/10.3390/nu16234192/s1; Table S1: Foods/drinks that make up the food groups shown in Table 3, labelled according to their likely contribution to a balanced diet; Table S2: Characteristics of children (included) with food records at each of three age (7, 10, and 13 years) compared with all other children recruited by the age of 7 years (excluded); Table S3: Distribution of percent energy contribution of free sugars from 3-day food records collected at ages 7, 10 and 13 years from children, with plausible energy intakes, taking part in the Avon Longitudinal Study of Parents and Children between 1999 and 2008; Table S4: Minimal model regression results (n = 729): predictors of being in the high free sugars (High-FS) compared with the low free sugars (Low-FS) group at all three ages (7, 10 and 13 years).

## References

[1] Scientific Advisory Committee on Nutrition (2015). Carbohydrates and Health.

[2] World Health Organization Guideline: Sugars Intake for Adults and Children. https://www.who.int/publications/i/item/9789241549028.

[3] Swan G.E., Powell N.A., Knowles B.L., Bush M.T., Levy L.B. (2018). A definition of free sugars for the UK. Public Health Nutr..

[4] Turck D., Bohn T., Castenmiller J., de Henauw S., Hirsch-Ernst K.I., Knutsen H.K., Maciuk A., Mangelsdorf I., McArdle H.J., EFSA Panel on Nutrition Novel Foods and Food Allergens (2022). Tolerable upper intake level for dietary sugars. EFSA J..

[5] Gracner T., Boone C., Gertler P.J. (2024). Exposure to sugar rationing in the first 1000 days of life protected against chronic disease. Science.

[6] Rennie K.L., Livingstone M.B. (2007). Associations between dietary added sugar intake and micronutrient intake: A systematic review. Br. J. Nutr..

[7] Amoutzopoulos B., Steer T., Roberts C., Collins D., Page P. (2020). Free and added sugar consumption and adherence to guidelines: The UK National Diet and Nutrition Survey (2014/15-2015/16). Nutrients.

[8] Fraser A., Macdonald-Wallis C., Tilling K., Boyd A., Golding J., Davey Smith G., Henderson J., Macleod J., Molloy L., Ness A. (2013). Cohort profile: The Avon Longitudinal Study of Parents and Children: ALSPAC mothers’ cohort. Int. J. Epidemiol..

[9] Boyd A., Golding J., Macleod J., Lawlor D.A., Fraser A., Henderson J., Molloy L., Ness A., Ring S., Davey Smith G. (2013). Cohort profile: The ‘Children of the 90s’—The index offspring of the Avon Longitudinal Study of Parents and Children. Int. J. Epidemiol..

[10] Emmett P. (2009). Dietary assessment in the Avon Longitudinal Study of Parents and Children. Eur. J. Clin. Nutr..

[11] Johnson L., Mander A.P., Jones L.R., Emmett P.M., Jebb S.A. (2008). A prospective analysis of dietary energy density at age 5 and 7 years and fatness at 9 years among UK children. Int. J. Obes..

[12] FAO/WHO/UNU (2001). Human Energy Requirements. Report of a Joint FAO/WHO/UNU Expert Consultation.

[13] Scientific Advisory Committee on Nutrition (2011). Dietary Reference Values for Energy.

[14] Cowan A.E., Jun S., Tooze J.A., Dodd K.W., Gahche J.J., Eicher-Miller H.A., Guenther P.M., Dwyer J.T., Potischman N., Bhadra A. (2023). A narrative review of nutrient based indexes to assess diet quality and the proposed total nutrient index that reflects total dietary exposures. Crit. Rev. Food Sci. Nutr..

[15] Northstone K., Emmett P. (2013). The associations between feeding difficulties and behaviours and dietary patterns at 2 years of age: The ALSPAC cohort. Matern. Child. Nutr..

[16] North K., Emmett P., Team A.S. (2000). Multivariate analysis of diet among three-year-old children and associations with socio-demographic characteristics. Eur. J. Clin. Nutr..

[17] Taylor C.M., Hays N.P., Emmett P.M. (2019). Diet at age 10 and 13 years in children identified as picky eaters at age 3 years and in children who are persistent picky eaters in a longitudinal birth cohort study. Nutrients.

[18] Perrar I., Schmitting S., Della Corte K.W., Buyken A.E., Alexy U. (2020). Age and time trends in sugar intake among children and adolescents: Results from the DONALD study. Eur. J. Nutr..

[19] Øverby N.C., Lillegaard I.T., Johansson L., Andersen L.F. (2004). High intake of added sugar among Norwegian children and adolescents. Public Health Nutr..

[20] Mok A., Ahmad R., Rangan A., Louie J.C.Y. (2018). Intake of free sugars and micronutrient dilution in Australian adults. Am. J. Clin. Nutr..

[21] Pawellek I., Grote V., Theurich M., Closa-Monasterolo R., Stolarczyk A., Verduci E., Xhonneux A., Koletzko B. (2017). Factors associated with sugar intake and sugar sources in European children from 1 to 8 years of age. Eur. J. Clin. Nutr..

[22] Langlois K., Garriguet D. (2011). Sugar consumption among Canadians of all ages. Health Rep..

[23] Hu F.B. (2013). Resolved: There is sufficient scientific evidence that decreasing sugar-sweetened beverage consumption will reduce the prevalence of obesity and obesity-related diseases. Obes. Rev..

[24] Ruxton C.H., Derbyshire E., Sievenpiper J.L. (2021). Pure 100% fruit juices–more than just a source of free sugars? A review of the evidence of their effect on risk of cardiovascular disease, type 2 diabetes and obesity. Nutr. Bull..

[25] HM Treasury Soft Drinks Industry Levy Comes into Effect. https://www.gov.uk/government/news/soft-drinks-industry-levy-comes-into-effect.

[26] Teng A.M., Jones A.C., Mizdrak A., Signal L., Genc M., Wilson N. (2019). Impact of sugar-sweetened beverage taxes on purchases and dietary intake: Systematic review and meta-analysis. Obes. Rev..

[27] Rogers N.T., Pell D., Mytton O.T., Penney T.L., Briggs A., Cummins S., Jones C., Rayner M., Rutter H., Scarborough P. (2023). Changes in soft drinks purchased by British households associated with the UK soft drinks industry levy: A controlled interrupted time series analysis. BMJ Open.

[28] Hashem K.M., Burt H.E., Brown M.K., MacGregor G.A. (2024). Outcomes of sugar reduction policies, United Kingdom of Great Britain and Northern Ireland. Bull. World Health Organ..

[29] Emmett P.M., Hays N.P., Taylor C.M. (2018). Antecedents of picky eating behaviour in young children. Appetite.

[30] Emmett P.M., Hays N.P., Taylor C.M. (2018). Factors associated with maternal worry about her young child exhibiting choosy feeding behaviour. Int. J. Environ. Res. Public Health.

[31] Fazzino T.L., Kong K.L. (2023). A new paradigm for investigating the etiology of obesity in early childhood: Exposure to added sugars and hyper-palatable foods in infancy and dysregulation of food reinforcement processes. Obes. Rev..

[32] Calabro R., Kemps E., Prichard I. (2023). Socio-cognitive determinants of sugar-sweetened beverage consumption among young people: A systematic review and meta-analysis. Appetite.

[33] Benton D., Young H.A. (2024). Early exposure to sugar sweetened beverages or fruit juice differentially influences adult adiposity. Eur. J. Clin. Nutr..

[34] Rose C.M., Birch L.L., Savage J.S. (2017). Dietary patterns in infancy are associated with child diet and weight outcomes at 6 years. Int. J. Obes..

