# Supplementary material for: The Contribution of Free Sugars to Energy Intake in Mid to Late Childhood: Comparisons Between Nutrient and Food Group Intakes and Antecedents of Diets High and Low in Free Sugars
Source: Nutrients. 2024 Dec 4;16(23):4192. doi: 10.3390/nu16234192 (PMC11644287; doi:10.3390/nu16234192)
Supplement: Supplementary file 1 [file nutrients-16-04192-s001.zip › nutrients-3341532-supplementary.pdf]

Supplementary Table S1. Foods/drinks that make up the food groups shown in Table 3, labelled according to their likely contribution to a balanced diet

Foods groups	Foods combined with each group	
All bread	White, brown, wholemeal, crackers, crispbreads	C
High fibre	Brown, wholemeal	C
Breakfast cereal	All types	C
Sugary cereals	Coco pops, Frosties etc.	M
Fat spreads	Butter, margarine all types, cooking fats and oils	D
Milk	Whole, semi-skimmed, skimmed	C
Other dairy	Cheese, yoghurt, cream, milk-based sauces	M
Sweet foods	Puddings, ice cream, cakes, buns, pastries, fruit pies, sweet biscuits, sweet flavourings for milk	D
Biscuits	All types of sweet biscuits	D
Confectionery	Sweets, fudge, mints, chocolates, chocolate bars, table sugar, honey, jam, marmalade, chocolate spread	D
Chocolate	Chocolate, chocolate bars	D
Savoury snacks	Potato crisps, corn-based snacks	D
Meat and poultry (plain)	Pork, beef, lamb, liver, chicken (roasted, grilled, stewed)	C
Meat (cured, coated, pies)	Sausages, meat pies and pasties, ham, salami, breaded chicken	M
Fish	White and oily fish, coated fish, fish fingers, shellfish	M

Vegetables	All plain cooked, canned & salad vegetables	C
Pulses	Baked beans, legumes	C
Potatoes (fried, roast)	Roast potatoes, fried chips, French fries, oven chips, vegetable dishes with pastry or coating	D
Plain potatoes	Boiled, mashed, baked potatoes	C
Pasta, rice, pizza	All rice, pasta, noddle and pizza dishes	C
Fruit	Citrus fruit, apples, bananas, soft fruit, canned and stewed fruit	C
SSSD	Sugar-sweetened soft drinks, diluted fruit flavoured drinks, lemonade, Ribena, cola drinks, sport drinks	D
Diet soft drinks	Artificially sweetened, diluted fruit flavoured drinks, diet cola, diet lemonade	D
Fruit Juice	Orange, apple, grape, pineapple, tomato	C

C, Core foods are nutrient-rich foods that are recommended as part of a balanced diet.

D, Discretionary foods are nutrient-poor foods which often contain excess fat or sugar and may unbalance a diet if consumed too often.

M, Mixed food groups contain some core and some discretionary foods.

Supplementary Table S2. Characteristics of children (included) with food records at each of three age (7, 10 and 13 years) compared with all other children recruited by the age of 7 years (excluded)

**Characteristic**	**Excluded (max. n=9721)**	**Included (max. n=4723)**	**P value**
Child sex			<0.001
Male	5094 (52.6%)	2325 (49.3%)	
Female	4591 (47.4%)	2395 (50.7%)	
Maternal education			<0.001
High	2284 (28.6%)	2094 (47.4%)	
Low	5691 (71.4%)	2321 (52.6%)	
Maternal age (years)			<0.001
<25	3028 (31.3%)	765 (16.2%)	
25-30	3045 (31.4%)	2172 (46.0%)	
>30	3612 (37.3%)	1783 (37.8%)	
Maternal BMI (kg/m^2)	23.0 (4.0)	22.7 (3.6)	0.005
Birthweight (g)	3381 (565)	3414 (548)	0.002

Values are n (%) or mean (SD).

Chi square test for categorical data or ANOVA for continuous data.

Supplementary Table S3. Distribution of percent energy contribution of free sugars from 3-day food records collected at ages 7, 10 and 13 years from children, with plausible energy intakes[a], taking part in the Avon Longitudinal Study of Parents and Children between 1999 and 2008

Age at food record (years)	≤5%	>5-10%	>10-15%	>15-20%	>20-25%	>25-30%	>30%	Total
7	5 (0.01%)	286(5.3%)	1485 (27.6%)	2035 (37.8%)	1147 (21.3%)	341 (6.3%)	86 (1.6%)	5385
10	17 (0.4%)	241 (5.4%)	1057 (23.7%)	1575 (35.3 %)	1099 (24.6%)	375 (8.4%)	95 (2.1%)	4459
13	18 (0.8%)	171 (7.7%)	560 (25.2%)	750 (33.8%)	462 (20.8%)	185 (8.3%)	75(3.4%)	2221

[a]Based on the ratio of reported energy intake to estimated energy requirement calculated individually considering sex, age and body weight at assessment with an increment for growth and physical activity.

Supplementary Table S4. Minimal model regression results (n=729): predictors of being in the high free sugars (High-FS) compared with the low free sugars (Low-FS) group at all three ages (7, 10 and 13 years)

Predictor variable	Reference category	Predictor category	Child in High-FS group at all three ages		
			**OR**	**95% CI**	**P value**
Child sex	Male	Female	0.76	0.56, 1.02	0.066
Maternal education	Low	High	0.97	0.71, 1.32	0.834
Maternal age (years)	25-30	<25	0.98	0.60, 1.61	0.948
		>30	1.03	0.74, 1.43	0.861
Maternal BMI	Normal	Underweight	0.77	0.46, 1.29	0.322
		Overweight/obese	0.70	0.47, 1.04	0.078
Birthweight (g)	2500-4000	<2500	**0.50**	**0.26, 0.96**	**0.036**
		>4000	1.04	0.65, 1.65	0.885

Low free sugars group (Low-FS) consumed ≤15% of energy from free sugars at each of the three ages.

High free sugars group (High-FS) consumed >20% of energy from free sugars at each of the three ages.

Total variance explained is 0.021 (Nagelkerke R square).
